# Supplementary material for: SMAD4 promotes somatic-germline contact during murine oocyte growth
Source: eLife. 2024 May 31;13:RP91798. doi: 10.7554/eLife.91798 (PMC11142639; doi:10.7554/eLife.91798)
Supplement: MDAR checklist [file elife-91798-mdarchecklist1.docx]

Materials Design Analysis Reporting (MDAR) Checklist for Authors

# For all that apply, please note where in the manuscript the required information is provided.

**Materials:**

| **Newly created materials** | **indicate where provided: page no/section/legend)** | **n/a** |
| --- | --- | --- |
| The manuscript includes a dedicated "materials availability statement" providing transparent disclosure about availability of newly created materials including details on how materials can be  accessed and describing any restrictions on access. | No datasets or material were created during this study. This is described in the Data Availability Statement preceding the Acknowledgements. |  |

| **Antibodies** | **indicate where provided: page no/section/legend)** | **n/a** |
| --- | --- | --- |
| For commercial reagents, provide supplier name, catalogue number and RRID, if available. | Methods |  |

| **DNA and RNA sequences** | **indicate where provided: page no/section/legend)** | **n/a** |
| --- | --- | --- |
| **Short novel DNA or RNA including primers, probes:** Sequences should be included or deposited in a public repository. | Primers used are listed in the Methods. |  |
|  |  |  |
| **Cell materials** | **indicate where provided: page no/section/legend** | **n/a** |
| **Cell lines:** Provide species information, strain. Provide accession number in repository **OR** supplier name, catalog number, clone number, **OR** RRID. |  | x |
| **Primary cultures:** Provide species, strain, sex of origin, genetic modification status. |  | x |

| **Experimental animals** | **indicate where provided: page no/section/legend)** | **n/a** |
| --- | --- | --- |
| **Laboratory animals** or **Model organisms:** Provide species, strain, sex, age, genetic modification status. Provide accession number in repository **OR** supplier name, catalog number, clone number, **OR** RRID. | Methods |  |
| Animal observed in or captured from the field: Provide species, sex, and age where possible. |  | x |

| **Plants and microbes** | **indicate where provided: page no/section/legend)** | **n/a** |
| --- | --- | --- |
| **Plants:** provide species and strain, ecotype and cultivar where relevant, unique accession number if available, and source (including location for collected wild specimens). |  | x |
| **Microbes:** provide species and strain, unique accession number if available, and source. |  | x |

| **Human research participants** | **indicate where provided: page no/section/legend) or**  **state if these demographics were not collected** | **n/a** |
| --- | --- | --- |
| If collected and within the bounds of privacy  constraints report on age, sex and gender or ethnicity for all study participants. |  | x |

| **Study protocol** | **indicate where provided: page no/section/legend)** | **n/a** |
| --- | --- | --- |
| If study protocol has been pre-registered, provide DOI. For clinical trials, provide the trial registration number **OR** cite DOI. |  | x |

| **Laboratory protocol** | **indicate where provided: page no/section/legend)** | **n/a** |
| --- | --- | --- |
| Provide DOI **OR** other citation details if detailed step- by-step protocols are available. |  | x |

| **Experimental study design (statistics details)** | | |
| --- | --- | --- |
| **For in vivo studies:** State whether and how the following have been done | **indicate where provided: page no/section/legend. If it could have been done, but was not, write not done** | **n/a** |
| Sample size determination |  | x |
| Randomisation |  | x |
| Blinding |  | x |
| Inclusion/exclusion criteria |  | x |

| **Sample definition and in-laboratory replication** | **indicate where provided: page no/section/legend** | **n/a** |
| --- | --- | --- |
| State number of times the experiment was replicated in laboratory. | Minimum three biological replicates, each containing multiple samples. |  |
| Define whether data describe technical or biological  replicates. | Biological replicates. All data has been included. |  |

| **Ethics** | **indicate where provided: page no/section/legend** | **n/a** |
| --- | --- | --- |
| **Studies involving human participants:** State details of authority granting ethics approval (IRB or equivalent committee(s), provide reference number  for approval. |  | x |
| **Studies involving experimental animals:** State details of authority granting ethics approval (IRB or equivalent committee(s), provide reference number  for approval. | All experiments were performed in compliance with the regulations and policies of the Canadian Council on Animal Care and were approved by the Animal Care Committee of the Research Institute of the McGill University Health Centre (RI-MUHC, protocol 7783) |  |
| **Studies involving specimen and field samples:** State if relevant permits obtained, provide details of authority approving study; if none were required, explain why. |  | x |

| **Dual Use Research of Concern (DURC)** | **indicate where provided: page no/section/legend** | **n/a** |
| --- | --- | --- |
| If study is subject to dual use research of concern regulations, state the authority granting approval  and reference number for the regulatory approval. |  | x |

3

| **Attrition** | **indicate where provided: page no/section/legend** | **n/a** |
| --- | --- | --- |
| Describe whether exclusion criteria were preestablished. Report if sample or data points were omitted from analysis. If yes report if this was due to attrition or intentional exclusion and provide  justification. | All samples and data points were included. |  |

| **Statistics** | **indicate where provided: page no/section/legend** | **n/a** |
| --- | --- | --- |
| Describe statistical tests used and justify choice of tests. | Methods and Figure legends. |  |

| **Data availability** | **indicate where provided: page no/section/legend** | **n/a** |
| --- | --- | --- |
| For newly created and reused datasets, the manuscript includes a data availability statement that provides details for access or notes restrictions on access. |  | x |
| If newly created datasets are publicly available, provide accession number in repository **OR** DOI **OR**  URL and licensing details where available. |  | x |
| If reused data is publicly available provide accession number in repository **OR** DOI **OR** URL, **OR** citation. |  | x |

| **Code availability** | **indicate where provided: page no/section/legend** | **n/a** |
| --- | --- | --- |
| For all newly generated custom computer code/software/mathematical algorithm or re-used code essential for replicating the main findings of the study, the manuscript includes a data availability statement that provides details for access or notes restrictions. |  | x |
| If newly generated code is publicly available, provide accession number in repository, **OR** DOI **OR** URL and licensing details where available. State any  restrictions on code availability or accessibility. |  | x |
| If reused code is publicly available provide accession number in repository **OR** DOI **OR** URL, **OR** citation. |  | x |

4

MDAR framework recommends adoption of discipline-specific guidelines, established and endorsed through community initiatives. Journals have their own policy about requiring specific guidelinesand recommendations to complement MDAR.

| **Adherence to community standards** | **indicate where provided: page no/section/legend** | **n/a** |
| --- | --- | --- |
| State if relevant guidelines(e.g., ICMJE, MIBBI, ARRIVE) have been followed, and whether a checklist (e.g., CONSORT, PRISMA, ARRIVE) is provided with the manuscript. | To the best of the authors’ knowledge, all guidelines have been followed. |  |
